# Attitude and Impact of Perceived Depression in the Workplace

**DOI:** 10.3390/ijerph110606021

**Published:** 2014-06-06

**Authors:** Yuan-Pang Wang, Clarice Gorenstein

**Affiliations:** 1 Institute & Department of Psychiatry (LIM-23), University of São Paulo Medical School, Rua Dr. Ovídio Pires de Campos, 785, São Paulo, SP 05403-010, Brazil; 2 Department of Pharmacology, Institute of Biomedical Sciences, University of São Paulo, Avenida Professor Lineu Prestes, 1524, São Paulo, SP 05508-900, Brazil; E-Mail: cgorenst@usp.br

**Keywords:** depression, workplace, cognitive symptoms, productivity

## Abstract

Information concerning the occurrence and consequences of depression in the workplace is scarce. This study estimates how workers perceive depression, to investigate depression-related disabilities, and management of depression in the workplace. This investigation is based on a cross-sectional web-based survey of 1,000 workers recruited from online sources. The participants were Brazilian workers, aged 16–64 years, current workers and managers, or who have worked within the past year. Subjects answered a 13-item questionnaire about depression, its related consequences in the workplace, and available resources to handle depression. Common symptoms attributable to depression were crying, loss of interest, and sadness. Almost one in five participants reported having ever been labeled by a doctor/medical professional as suffering from depression. However, the majority of ever-depressed workers (73.5%) remained working. Performance-related impairments were reported by around 60% of depressed workers who continued working. Over half of them also complained about cognitive symptoms (concentration difficulties, indecisiveness, forgetfulness). One in three workers had taken off work due to depression (mean 65.7 out-of-role days), with these periods being lengthier for men than women. Managers underestimated the number of days out-of-role (29.5 days). The findings suggested that identification and management of symptoms of depression should be set as a priority in worker’s health care.

## 1. Introduction

Neuropsychiatric disorders exact a weighty toll on individuals, family, and society [1]. Mental disorders are associated with lost working time, accounting for 15.4% of the total disease burden in market economies [2]. Although depression has become the leading cause of disability worldwide [2], studies investigating the influence of depression in the workplace are scarce. Data of work performance and loss productivity that combine figures of expenditure, disability, morbidity, mortality, and accident injury are regarded as a major gap to advance on current health knowledge [3].

Disability can be expressed by impairments, activity limitations, and participation restrictions, reflecting the interactive relationship between individuals with a health problem and personal and environmental factors [4]. Lost workdays stands as an important component of disability, which can be directly measured by missed workdays (absenteeism). Conversely, non-quantifiable low performance while at work (presenteeism) remains in the shadow, under the stigma of mental disorder and the fear of losing jobs in periods of economic crisis [5]. Pooled data of 24 participating countries of the WHO Mental Health survey [6] have indicated that 12.8% of respondents reported one or more days totally out-of-role due to health conditions per year, with a median of 51.1 workdays. Mental disorders stood out as responsible by a large proportion of the number of days out-of-role, indicating the pressing need of addressing their impact on the economy. Lost productive time of depression *vs*. without depression among workers represents 5.6 h/week *vs*. 1.5 h/week [7,8]. Every year, the US employers expend over USD$44 billion in depression-related lost productivity [8]. A higher figure is found in a recent survey in Europe [9], where the cost of depression due to lost productivity is projected at £77 billion annually.

The regional report of the Global Burden of Diseases (GBD) [10] has specified that eight out of the 10 leading causes of Years Living with Disability (YLD) in the Tropical Latin America region are either chronic pain or mental disorders. Despite of the sense pointed by this trend, far-reaching data of work-related depression in Latin America and Caribbean (LAC) region are still limited. Results from the first GBD report in Brazil [11] showed that neuropsychiatric disorders ranked first among the major causes of YLD (34%), followed by chronic respiratory diseases (11.2%).

Deemed as a new advanced economy country, Brazil is facing an epidemiologic transition characterized by an increasing burden of chronic non-communicable diseases surpassing infectious diseases [12]. Most previous Brazilian health studies were based on administrative official workers’ compensation databases [13] obtained from the National Institute for Social Security (INSS). According to INSS, five out of the 10 leading causes of compensation benefits are mental disorders, accounting for 19% of total cost with disability benefits [14]. These figures are misleading because Brazilian labor laws require 15 missed workdays (absenteeism) before being entitled to request compensation benefits (sick leave), what omits the cases of sickness-related absences of less than 15 days. In addition, the magnitude and effects of presenteeism remain largely unknown in this middle-income LAC country.

Recently, Yano and Santana [15] reported that one-year prevalence of workdays lost due to health problems affected 12.5% of Brazilian industrial workers, with 5.5% being directly attributed to work and 4.1% being aggravated by work. In results from a large survey conducted in a metropolitan area [16], 13.1% of the general population reported at least one day totally out-of-role in the month prior to the interview, with a median of 41.4 days out-of-role per year. Chronic pain and mood disorders were the two conditions of highest impact after controlling for age, sex, education, employment, and comorbidity [17]. However, existing studies have not provided specific information on the illnesses associated with workday loss and no broader study was found to investigate depression in the workplace in Brazil. Further, understanding how depression is managed can aid to de-stigmatize its perception and reformulate protective labor laws for those affected individuals.

In current survey, we asked to workers on common symptoms of depression and their impact on labor performance. The objectives were: to investigate how the workers perceive the occurrence of depression; to estimate the depression-related disabilities; and how depression is recognized and managed in the companies.

## 2. Methods

This is a web-based cross-sectional survey, known as “netquest”, conducted in accordance with guidelines of the International Chamber of Commerce and European Society for Opinion and Marketing Research (ICC/ESOMAR) [18]. The current study is a Brazilian counterpart of a larger multicentric survey [9], the Impact of Depression in workplace in Europe Audit (IDEA) launched by the European Depression Association (EDA) [19].

### 2.1. Sampling and Recruitment

The recruitment of the respondents was multisourced, closed, and “by invitation only”. This procedure followed ESOMAR’s quality controls to guarantee unique participants and to avoid duplications or fraud, which is based on established agreements with validated Internet sites. The partners were mostly Internet portals, service providers, online stores, airlines, communities, *etc*., with audited customer databases. People invited in this study were Internet users who have joined our online panel. As we could not target “people who must be currently or have been previously employed”, we have selected a general population sample. Therefore, no criteria were taken into consideration, except for age stratum from 18 to 65 year-old. This approach offered the possibility to engage a broad range of socio-demographic profiles by natural fallout, in order to select a random sample of respondents. For targeting the sample, there is a random extraction on needed demographics and quotas structure on age, gender and region. After receiving an invitation email for the online panelists, the participant was allowed to fill out only one form. Personal data were checked and possible duplicates removed. No quotas on company size were applied.

The planned sample size of 1,000 participants met the standard of national representativeness for marketing and opinion research. After data collection, the crude results were weighted to ensure the eligible individuals were representative of the target profile: Brazilian workers, aged 16 to 64 year-old, current worker and manager, or have worked and managed within the last 12 months.

### 2.2. Instrument

A 13-item questionnaire was elaborated by members of the European Association of Depression [19] to investigate the Impact of depression in the workplace and has been applied in over 7,000 European workers [9]. This instrument was translated and adapted for use in Brazilian-Portuguese speaking respondents.

All respondents answered the online questionnaire after proper identification of sex, age, marital status, working status and position of manager, size of company (small, medium, and large), highest educational level, and dwelling region in Brazil (North, Northeast, Middle-East, Southeast, and South). The questionnaire determined if the participant was currently working or was previously employed in the past 12 months, otherwise the survey would be halted. Also, those respondents aged outside the range of 16 to 64 years were not allowed to proceed in the questionnaire.

First, the participants have to score health conditions from the least disabling (1) to the most disabling one (5). The five listed health problems were: heart/blood pressure/circulation problem; hearing loss/deafness; depression; alcoholism/alcohol abuse; and cerebrovascular disease [Q1].

Following, they have to choose four among a list of 10 items (nine symptoms/behaviors plus one open-ended item to include other possibilities) that could indicate someone in the workplace is depressed and indicate four attributes/symptoms associated with depression in general [Q2]. At this point, the participants were asked if a doctor or medical professional has ever diagnosed them as having depression [Q3]. If positive, they indicated the symptoms they experienced among a 10-item list and whether they continued to work in the last time they had depression [Q4].

Using a 0-to-10 scale, from the worst to the best functioning, the respondents have to rate the following: (a) the usual performance of the workers holding a similar position; (b) their usual performance during the past year when they did not experience depression; and (c) their overall job performance when they had depression the last time. From a list of nine items, those depressed respondents reported which behaviors they demonstrated more than usual while still working and which attributes/symptoms most impacted their usual performance in the work [Q5].

The question “have you ever taken time off work because of your depression?” was asked to every ever-depressed workers [Q6]. If affirmative, they answered the number of working days that have had to take off during the last time of depression, the attributes/symptoms that had caused it, and the reasons for taking time off [Q7]. If the worker had omitted mentioning depression, the reasons of non-disclosure were recorded.

In the last section, all workers reported if there were any colleagues at their workplace that ever had depression [Q8], how they came to know about the depression of the colleague, and what they did about this [Q9]. Considering average days of “sick leave” per year, the respondents should estimate how many days were attributed to depression [Q10]. If the respondent was a manager, they also answered on available resources to support depressed employees and how convenient was the support [Q11]. Finally, all respondents indicated the general impact of depressive symptoms on productivity [Q12] and what kinds of provision would be useful for depression [Q13].

### 2.3. Confidentiality

All participants must accept the general terms and the privacy policies prior to complete their registration. The netquest was conducted in accordance with codes and guidelines of the ICC/ESOMAR [18], which were explicitly in force in Brazil since 2009.

### 2.4. Statistical Analysis

The final results were weighted to correct for minor discrepancies by using the random iterative method (RIM) [20] to adjust for the distribution of demographic profile of Brazilian workers, such as age, gender, region and working status. Thereafter, data were subjected to descriptive analysis. Usual hypothesis testing such as chi-square and ANOVA was adopted to contrast difference between subgroups of the sample, respectively for categorical and continuous data. Data bank was split whenever we contrast opinion between managers and employees. All analyses were conducted through SPSS version 21.0 software [21]. The level of significance of 0.05 was considered for 2-sided tests.

## 3. Results

### 3.1. Demographics

Demographic characteristics of the sample are described in Table 1. Briefly, the sample of 1,000 workers was made up of 57.3% male and 42.7% female participants, with a mean age of 36.8 years (standard deviation [SD] 11.6, range 18 to 64 years). Most of participants were in the age bracket of 25–34 years, married, low educational level (up to 8 years), medium income per month, and residing in South Brazil.

**Table 1:** Demographic characteristics of the participants (*n =* 1,000).

Variable	*n*	Weighted Proportion
Gender (%)		
Male	589	57.3%
Female	411	42.7%
Mean age (yo., SD)		36.8 yo. (11.6)
Age bracket (%)		
18–24 yo.	162	17.4%
25–34 yo.	307	30.1%
35–44 yo.	259	25.3%
45–54 yo.	196	19.2%
55–64 yo.	76	8.0%
Marital status (%)		
Married	423	41.2%
Cohabiting	176	17.8%
Single	329	33.2%
Separated or divorced	62	6.7%
Widowed	6	1.1%
Educational level (%)		
Up to 8 years	764	76.4%
8 to 11 years	226	22.6%
12 or more years	10	1.0%
Region of Brazil (%)		
North	75	8.0%
Northeast	224	23.8%
Southeast	129	7.1%
South	414	44.6%
Middle-West	158	16.4%
Income per month (%)		
low †	44	4.5%
medium ††	632	65.2%
high †††	262	24.3%

yo.: year-old; SD: standard deviation; † Up to Brazilian Real (BRL$) 1000; †† BRL$ 1001 to BRL$ 5000; ††† BRL$ 5001 or more; Missed data were omitted in the tabulation.

Most of participants (79%) were full-time workers, 17% part-time, and 4% was previously employed in the past 12 months. Regarding working position, 900 were employees and 100 managers of a total of 100 companies, 20 from small size (1–50 employees), 28 from medium (51–250 employees) and 52 from large (more than 250 employees) companies.

### 3.2. Perceived Depression

Overall, depression was considered by 1,000 workers as the third most disabling health problem (18%) to perform daily activities, being significantly less disabling than cerebrovascular disease (31%; χ^2^ = 373.28, *p* < 0.0001) and alcohol-related problems (26%; χ^2^ = 383.87, *p* < 0.0001). In contrast, depression was regarded more disabling than heart disease/hypertension (14%) and deafness/hearing loss (11%). The symptoms more attributable to depression were: crying without reason (69%), loss of interest (68%), and low mood or sadness (63%). Subsequently, sleep problems (50%), weight and appetite change (38%) were less frequent. The cognitive dysfunctions, considering the items of difficulty of concentration, or indecisiveness, or forgetfulness, were viewed by 34% of the workers as relevant indicators of depression (Table 2).

The most reported signs and behaviors of depression in the workplace were: withdrawing from colleagues (73%) and crying at work (70%). Making more mistakes than usual (42%), sick leave (33%), and taking more time to complete jobs (31%) were also salient single behaviors that indicated depression. Joint performance-related items (more mistakes than usual, more time to complete jobs, missing deadlines, and falling asleep at work) were viewed by 70% of workers as indicative of depressive behaviors. Cognitive symptoms (indecisiveness and forgetfulness) accounted for 39% of the signs of depressive behavior at work (not shown).

**Table 2:** Common depressive symptoms as described by all workers (*n =* 1000); in the most recent episode; with impact on work performance while depressed; and which caused taken off work.

Depressive symptoms	Symptoms Indicating Depression	Symptoms Last Time had Depression	Symptoms with Impact on Performance	Disabling Symptoms causing Taken off Work
	*n* = 1,000	*n* = 189	*n* = 139	*n* = 63
Low mood or sadness	63%	71%	52%	62%
Loss of interest in daily activities	68%	71%	59%	64%
Trouble sleeping/insomnia	50%	57%	44%	57%
Crying for no reason	69%	55%	28%	51%
Changes in weight and appetite	38%	47%	30%	21%
Difficulty planning day to day activities	25%	36%	28%	26%
Trouble concentrating	21%	35%	36%	36%
Indecisiveness	9%	27%	13%	8%
Forgetfulness Other symptoms	9% <0.1%	24% 6%	19% 5%	21% 0%
Cognitive symptoms *	34%	53%	53%	50.8%

* Cognitive symptoms encompass: trouble concentrating, indecisiveness, and/or forgetfulness.

**Figure 1:**
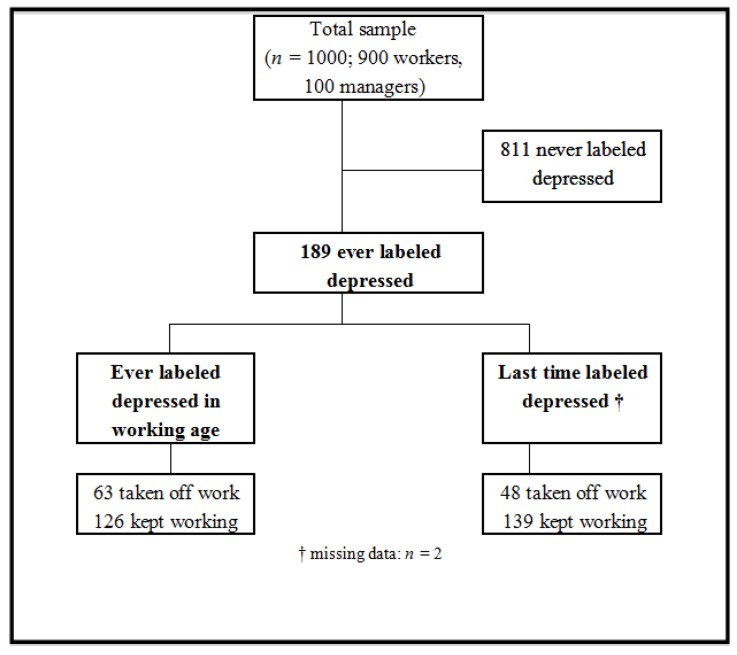
Survey chart for participant workers (*n =* 1,000).

Almost one in five participants (Figure 1) reported ever being labeled by a doctor/medical professional as having depression (*n =* 189), with a male/female sex ratio of 1:1.8. During the last period of reported depression, the most experienced symptoms among those ever-depressed workers (Table 2) were: low mood or sadness (71%) and loss of interest (71%). Then, sleep problems (57%), crying (55%), and weight and appetite changes (47%) were reported. Cognitive dysfunctions, considering the items of difficulty of concentration, or indecisiveness, or forgetfulness, accounted for 53% of ever-depressed ones. Considering gender, women reported significantly more crying (70% *vs*. 35%, χ^2^ = 24.33, *p* < 0.0001) and loss of interest (82% *vs*. 57%; χ^2^ = 10.08, *p* < 0.0001) than men. Regardless of experiencing or not previous depression, the overall perception of depressive symptoms was not significantly different (χ^2^ = 0.024, *p* = 0.88).

### 3.3. Impact of Perceived Depression in the Workplace

Since the period they started working, one-third of the 189 ever labeled depressed workers had taken time off work. A smaller proportion of 24.3% reported that they had taken off work during the last time they had been labeled depressive. The great majority (66.7% to 73.5%) remained working in contrast to those who have stopped working because of depressive symptoms, irrespective of depressed period and/or gender (Figure 1).

Among those who continued working when were labeled depressed last time (*n =* 139), 68% reported withdrawing from colleagues, 40% taking more time to complete jobs and indecisiveness, and 37% making more mistakes and crying as typical symptoms of depression. Overt performance-related impairments, such as taking more time to complete jobs, making more mistakes, and/or missing deadlines, were reported by around 60% of those depressed whom continued working.

Among the attributes/symptoms that had impacted on the ability to perform tasks at work (Table 2), the most troublesome were: loss of interest (59%), low mood or sadness (52%), sleep problems (44%), and trouble concentrating (36%). Further cognitive symptoms impacted for 53% of depressed workers.

**Figure 2:**
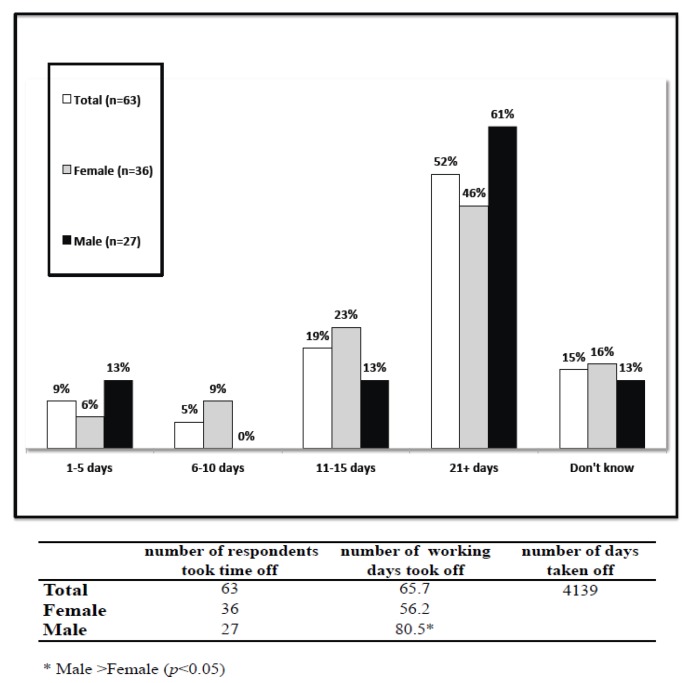
Average number of days taken off work because of depressive symptoms, all workers who taken off work (*n* = 63) and by gender.

Reflecting ever labeled depressed individuals, a total loss of 4,139 working days per year was reported by the 63 individuals who took off work because of depression. Absenteeism calculated considering the part-time workers (*n =* 11) was 3,795 days. The mean number of days out-of-role was 65.7 days, being significantly lengthier for men than women (80.5 *vs*. 56.2; *F* = 4.21, *p* = 0.045). More than half of these workers took off 21 or more days (Figure 2).

Common attributes/symptoms directly associated with taking off work were loss of interest (64%), low mood or sadness (62%), sleep problems (57%), crying (51%), and trouble concentrating (36%). The cognitive symptoms impacted for 50.8% of the workers (Table 2). Ninety percent of workers whom self-rated their job performance as 8–10 (the top performance) when was not depressed, subsequently downward rated (<8) their performance in the last time when became depressed.

Among those who ever took off work because of depressive symptoms (*n =* 63), only 7% spontaneously disclosed that the taking time off work was linked to depressive symptoms, whereas 71% revealed the problem upon medical indication and 15% omitted the reason of the need of taking time off work. The key reasons for non-disclosure were the fear of putting their job at risk or because they considered it a private issue.

### 3.4. Recognition and Management of Depressive Symptoms

Almost half of the participants were not able to recognize probable depressed workers. Most of the participants know (34%) or believe (19%) that someone at work has had depression, while one-third don’t know and 14% stated that no one has had depression in their workplace setting. The proportion of managers *versus* employees that could identify depressed people at workplace was significantly higher (57% *vs*. 32%; χ^2^ = 25.36, *p* < 0.0001). However, the awareness was more frequently associated to self-disclosure by the depressed worker (42%).

Helpful and supportive behaviors were frequently observed among managers whereas employees tended to remain neutral or showed no helping attitude. Among those who know/believe that someone at work has had depression, 53% discussed and/or offered help, and 45% encouraged seeking after professional care. Conversely, 13% didn’t know how to react/what to say, 12% did nothing, 7% avoided talking to them about, and 5% warned that depression must not affect their work.

The available sources of help for a manager in dealing with depressed employees were: human resources department (50%), and medical professional (42%). In contrast, 24% reported no formal support or resources in their company. The quality of support received from the company to handle depressed employees was evaluated as very good by 17% and fairly good by 44%. On the other hand, 25% of the managers refrained of answering this question.

In general, there was agreement between managers and employees on the necessity of more supportive resources to deal with depression in workplace, such as: counseling services (84%), training for all employees (39%) and human resources team (34%), and better government legislation/policies to protect employees (30%). Probably training managers and educational leaflets or brochures also might contribute to benefit depressed working population.

Concerning the direct impact of someone at workplace suffering from depressive symptoms, most respondents think that those employees would be significantly less productive (66%; *p* < 0.0001), would take extended sick leave (36%; *p* < 0.001), and would affect the mood of all employees (31%; *p* < 0.001). Only 6% answered that depression would not have any impact.

Despite the widespread belief that depression can have a negative impact on productivity, most of managers (62%) don’t know how many days per year people at their work take off because of depression. They estimated that the average number of working days out-of-role as 29.5 days, while the actual period of leave was more than double that figure. Therefore, most of companies’ executives ignore the cost of depression to their business.

## 4. Discussion

Depression in the workplace affects several domains such as work productivity, environment climate, with negative consequences to countries’ economy. The personal and societal burden of this issue cannot be neglected when we become aware of the proportion of affected people. In current attitude and opinion survey, around one in five Brazilian workers reported has received a diagnosis of depression in their lifetime. The validity of rates of this web survey (18.9%) finds support in recent prevalence study conducted with probabilistic technique in Brazilian general population, which reported a similar rate of 19.1% [22], and in high-income countries, such as the US (19.2%) and France (21.0%) [23]. Likewise, one in four (26%) employees in the UK reported a depressive episode throughout their working age [9,19]. A web survey in Brazilian general population reported 10.5% of depression in the past 12 months [24]. Also, the median age of onset of depression was concentrated among those individuals in their late twenties, when most people should be economically productive [22].

In competition-based modern societies, depression is highly prevalent [23] and is perceived by workers as a moderate-to-severe condition, with harsh impact in work performance. This has just confirmed old news: individuals with depression reported more impairment in productivity than those without depression [24,25]. Also, it is clear from the worker’s judgment that overt behaviors (such as crying, withdrawal from colleagues, *etc*.) were viewed as indicators of depression in the workplace, which were regarded as important as the symptoms of loss of interest and sad mood. This stereotyped opinion indicates a misconception, as far as both the core features of depression and non-specific behavioral depressive signs were identified at similar frequency. Ever-depressed ones viewed the impact of these overt symptoms on the work performance as just mild. Some covert symptoms of depression also affect the people in their workplace, such as less recognizable cognitive symptoms.

Indecisiveness, attention and memory deficits are a set of common depression-related cognitive symptoms in the workplace. These difficulties are much more frequent than previously thought [26,27,28]. Over half of ever-depressed participants reported these covert cognitive impairments as severe symptoms of depression, which intensity is disabling enough to impact daily routine [29]. Additional studies showed evidence of cognitive deficits associated with depression leading to substantial work impairment, performance dysfunction, and loss of employment [30,31,32]. In extreme cases, these symptoms can cause taken off work. Recent evidence has demonstrated that these deficits may manifest and persist independently of the core depressive symptomatology of sadness and loss of interest [33], but the degree of cognitive dysfunctions cannot be accounted by the severity of depression in some patients [26]. Particularly, executive function, working memory, attention, and psychomotor processing were associated with diminished productivity as whole [26,34]. Therefore, addressing cognitive dysfunction may have vital therapeutic implication in the workplace.

The negative effect of depression on productivity is a matter of concern. Absenteeism, as expressed by the mean number of days taken off per year, was high and lasted on average more than two months. This figure was about twice higher the number of days estimated by the managers and large-scale studies in general population considering all health conditions [6,17]. Although measuring presenteeism is challenging [24], self-rated reduction in performance plus cognitive impairments might be a warning indicator of depression. Most sick leaves can be prevented if there are better policies in the companies to deal with milder depressed employees in early stage of disability.

The findings of our study are in line with the literature in relation to the higher likelihood of women suffering from depression than men [35,36,37], however, men take longer time than women to return to work. Higher prevalence of depression in women does not explain this conflicting finding of lengthier recovery among men. Depression in males is often misunderstood and misdiagnosed: while women are used to express their feelings [38], men tend to demonstrate behavioral changes when depressed [39]. At the symptom level, psychomotor functioning and cognitive impairments appear to affect unequally both genders [40,41,42]. Men are unwilling to self-perceive themselves as depressive, unless they experience severe symptom patterns. Neglected in the psychiatric literature, the symptoms of depression in male are usually more covert or “masked” [43]. Hence, when depression strikes male workers, their connection at the feeling level tend to be poorer than at the behavioral level. By masking depression as secret distress in the workplace, many depressed male workers go unrecognized and untreated, although deeply depressed [24].

The reasons of this unfavorable aftermath should also be interpreted in light of illness behavior and help-seeking habits of men [44,45,46]. Gender-related attitudes may shape opinions towards depression [47]. In addition to gender-determined psychopathological difference when depressed [38], men show less favorable help-seeking attitudes concerning their health needs [48,49]. Disregarding job strain, the propensity to keep working instead of seeking help is a commonplace [50] and could subsidize their underutilization of mental health services. Recent analyses of occupational compensation data in Brazil, male workers were the group that showed the highest health burden [13,51]. As consequence of delayed care and chronic course, the time to recover from residual depressive symptoms among male workers tends be lengthier [52,53]. Therefore, male workers should be encouraged to be aware of the common depressive symptoms and actively request early treatment/advice. Appropriate tools for coping with and healing depression should be offered to men. This scenario suggests the need of educational programs targeting to improve men’s willingness to contact specialty mental health services.

The observation that most of depressed workers remained in the job is a big warning shot. The effects of presenteeism on labor performance should be judiciously considered in the companies. Managers and workers who were not capable of recognizing and helping those individuals suffering from depressive symptoms indicate a window of opportunity for preventive actions, such as early identification, proper referral, and adequate resource provision. Colleagues should offer proactive help, in an effort to promote awareness of mental disorders and minimize the fear of being dismissed in time of global economic instability. Implementing labor-specific health policy and setting worker’s protection laws would alleviate negative consequences of this situation. Provided that the productivity could be improved among workers who attained wellness with early treatment [34], this fact represents a wake-up call for a greater collaboration between companies and mental health researches to bringing back those with depression to the workplace.

Some cautions should be born in mind in the data interpretation before generalizing the findings to the Brazilian workforce. The netquest is an increasingly adopted investigation tool in social and marketing research, with the advantage of being an efficient fast survey method. However, self-reported information obtained from netquest using non-validated or standardized instruments for diagnosis of depression should be cautiously compared with surveys conducted with in-person interviews. Web surveys could allow emerging faked answers or incomplete questionnaires. The representativeness of the recruited sample is unclear. As the coverage of Internet service does not apply equally to all population strata in non-developed countries, disadvantaged individuals who don’t have electronic access might be underrepresented in the sample, e.g., informal or rural workers. Also, older workers from the 55 to 64 years of age bracket would be less liable than the younger strata to participate in such web surveys due to their lower intrinsic familiarity with Internet tools. Regular exclusion of workers over 65 years could have biased the representativeness of active working population, due to the recent phenomenon of delayed exit from the Brazilian labor market. Finally, people with severe cognitive symptoms may have not participated of this survey, as their employment rate is probably low, thus they may not be easily recognizable in this sample.

Recall bias might have occurred because the workers self-reported subjective experience of depression in a past timeframe, producing distortions in the accuracy or completeness of the retrieved recollections. Reported depression during the working age might have started when participants were not working yet or while they were unemployed, as there was no rigorous assessment with standard instruments.

## 5. Conclusions

The results of this survey draw attention to the negative consequences of depressive symptoms in the workplace. A large fall in productivity may result from the performance-related impairments reported by workers who continued working despite displaying symptoms. Additional loss comes from the periods of sick leave, where the number of absent days is much longer than estimated by both managers and workers.

Tackling the manifestations of depression and implementing cost-effective policies to handle its deleterious effects should be set as a priority research and clinical agenda for the health of people in the labor market. At the level of the country’s economy, data of the current investigation in Brazilian workforce can serve as comparison to other LAC countries at similar stage of economic development. Labor-based societies should dispel the persistent stigma about depression in occupational settings. Changes in attitudes of the sufferers of depression, co-workers, and the manager or boss can shorten the delay to obtain treatment, reducing the impact to the individual, the society, the company, and the economy as whole. Granted resources shall help individuals to seek advices for this widespread problem in the workplace. These ingredients might be the first footstep of a large nationwide campaign to fight against depression in the workplace. Depression-free workers can serve as a proxy of a nation’s productivity, a valuable asset that may ultimately render stronger its economy.
